# Studying micro RNA Function and Dysfunction in Alzheimer’s Disease

**DOI:** 10.3389/fgene.2012.00327

**Published:** 2013-02-06

**Authors:** Walter J. Lukiw, Tatiana V. Andreeva, Anastasia P. Grigorenko, Evgeny I. Rogaev

**Affiliations:** ^1^Department of Neurology, LSU Neuroscience Center, Louisiana State University Health Sciences CenterNew Orleans, LA, USA; ^2^Department of Ophthalmology, LSU Neuroscience Center, Louisiana State University Health Sciences CenterNew Orleans, LA, USA; ^3^Department of Genomics and Human Genetics, Russian Academy of Medical Science, Vavilov Institute of General GeneticsMoscow, Russia; ^4^Department of Psychiatry, Brudnick Neuropsychiatric Research Institute, University of Massachusetts Medical SchoolWorcester, MA, USA; ^5^Faculty of Bioengineering and Bioinformatics, Lomonosov Moscow State UniversityMoscow, Russia

**Keywords:** aging, Alzheimer’s disease, amyloidogenesis, inflammation, miRNA, neurotrophism, presenilin, synaptogenesis

## Abstract

Alzheimer’s disease (AD) is a tragic, progressive, age-related neurological dysfunction, representing one of the most prevalent neurodegenerative disorders in industrialized societies. Globally, 5 million new cases of AD are diagnosed annually, with one new AD case being reported every 7 s. Most recently there has been a surge in the study of the regulatory mechanisms of the AD process, and the particular significance of small non-coding ∼22 ribonucleotide RNAs called micro RNAs (miRNAs). Abundant data have profiled miRNA patterns in healthy, aging brain, in mild cognitive impairment (MCI), and in the moderate- and late-stages of AD. The major mode of action of miRNA is to interact, via base-pair complementarity, with ribonucleotides located within the 3′ untranslated region (3′-UTR) of multiple target messenger RNAs (mRNAs), and in doing so decrease the capability of that specific mRNA to be expressed. Many miRNAs are highly cell- and tissue-specific. The human brain appears to use only a highly specific fraction of all known human miRNAs, whose speciation and complexity are defined as a discrete subset of all known small non-coding RNAs (sncRNAs) in the brain. In general, in contrast to normally, aging human brain, in AD a family of pathogenically up-regulated miRNAs appear to be down-regulating the expression certain brain-essential mRNA targets, including key regulatory genes involved interactively in neuroinflammation, synaptogenesis, neurotrophic functions, and amyloidogenesis. These up-regulated, NF-kB-sensitive miRNAs, involved in the innate immune and inflammatory response and synaptic, neurotrophic, and amyloidogenic functions include miRNA-9, miRNA-125b, miRNA-146a, and miRNA-155. Other miRNAs of the miRNA-15/107 family, miRNA-153 and miRNA-190, and others, will be discussed. Overall, this manuscript will review the known contribution of miRNAs to aging brain function and the role they appear to play in the incidence and progression of AD.

## Introduction

Alzheimer’s disease (AD) represents the most common type of intellectual impairment and memory loss of the aged, characterized by the progressive erosion of cognition, functional ability, coordination, mood, behavior, and memory (Alzheimer et al., 1995; Alzheimer Association, 2012). As AD advances, a lifetime of learning, skills and memory is progressively lost. At the one hundred sixth anniversary of Alois Alzheimer’s (1864–1915) first description of AD (in 1906), a tremendous amount of scientific research effort into this common neurological disorder has been obtained, however many problematic gaps in our knowledge still remain. The increasing life expectancy and demographics of our aging population cast significant healthcare concerns over the future management of this leading cause of intellectual loss and cognitive decline. There are currently no curative nor effective clinical treatments for AD, and pharmacological strategies directed at AD symptoms, and specifically targeted to the insidious, progressive, and inflammatory nature this brain degeneration, have met with extremely disappointing results (Lukiw, 2007a, 2008, 2012a; Vellas et al., 2007; Fisher Center for Alzheimer’s Research Foundation, 2011). In this second century of AD research, more objective study, novel research investigations and interpretations and alternative mechanistic hypotheses are clearly required to more successfully address this complex and expanding health care concern. This review deals with novel observations of micro RNA (miRNA), a relatively recently described epigenetic regulatory control mechanism, on shaping the gene expression patterns in AD brain versus healthy age-matched controls.

## Human microRNA

**Figure 1:**
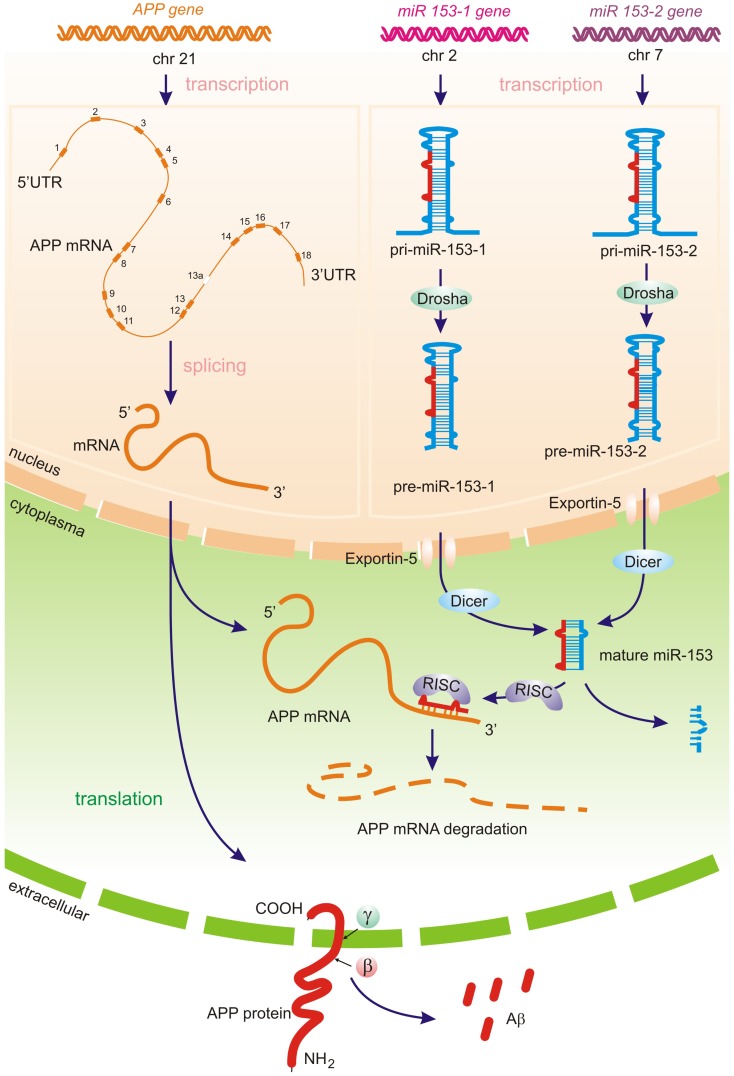
**Predicted miRNA processing pathways – APP translation and amyloid beta generation may potentially be directly regulated by brain-specific miRNA-153 family**. Primary miRNA-153-1 and miRNA-153-2 transcripts (pri-miR) produced by RNA polymerase II, is then cleaved by the Drosha complex in the nucleus. The resulting hairpins pre-miR-153-1 and pre-miR-153-2 are transferred by Exportin-5 to the cytoplasm, were the Dicer-complex generates the mature biologically active miR-153. The one strand of the miR-153 in the RISC complex (RNA-induced silencing complex) represses APP translation through APP 3′-UTR mRNA cleavage, whereas the other miRNA strand is degraded. Decreasing of miR-153 levels may lead to increased APP expression (Long et al., 2012) and further amyloid beta production.

Micro RNAs represent a class of short regulatory RNAs, which may promote sequence-dependent degradation of target messenger RNAs (mRNAs) or suppress translation of corresponding protein. The role of miRNAs in many fundamental functions including neurodevelopment, plasticity, and apoptosis as well as in psychiatric disease has been reported (Kosik and Krichevsky, 2005; Bredy et al., 2011). Mature ∼19–24 nucleotide (nt) RNAs are derived from longer miRNA precursor molecules – primary miRNA transcript (pri-miR) ∼70–100 nt with a stem loop structure. The pri-miR is cleaved within the nucleus by a protein complex called Drosha into a pre-miR, which is then shuttled to the cytoplasm by the Exportin-5 pathway. In the cytoplasm, the pre-miRNA is cleaved into mature miRNA with 3′ overhangs by the Dicer endonuclease. One of the two strands of miRNA in the RISC complex binds to the 3′-UTR of target mRNAs in a sequence-dependent manner, resulting in posttranscriptional silencing by translational repression or/and mRNA degradation (Figure 1) miRNAs have been shown to form heteroduplexes not only with the 3′-UTR but also with protein-coding regions as well as the 5′-UTR (Orom et al., 2008; Tay et al., 2008).

The biological role of miRNAs has previously been reported in multiple diseases, including AD, and in the last several years studies have shown several miRNAs to be either up- or down-regulated in AD, as well as their ability to regulate several important AD genes.

## Alzheimer’s Disease – An Expanding Healthcare Concern

An estimated 5.4 million people in the United States have AD, and healthcare treatment for AD patients in the United States currently involves a staggering 15 million unpaid caregivers and 183 billion dollars in annual costs (Alzheimer Association, 2012). The projected yearly expense of AD healthcare is estimated to soar to 1.1 trillion dollars by the year 2050 (Lukiw, 2007a, 2008, 2012a,b; Fisher Center for Alzheimer’s Research Foundation, 2011; Alzheimer Association, 2012). Currently, this places a tremendous socioeconomic burden on both AD caregivers and an already strained healthcare system. In the foreseeable future the prognosis for AD incidence and soaring medical costs become even more stark and overwhelming and in the next 10 years will outstrip our economic and healthcare capacity to deal with this disease (Lukiw, 2007a, 2008, 2012a; Fisher Center for Alzheimer’s Research Foundation, 2011; Alzheimer Association, 2012). Importantly, our increasing life expectancy and the demographics of our aging population, both domestically and on a global scale, cast significant concerns over our medical and socioeconomic capability to effectively manage this rapidly expanding neurodegenerative disease. Currently, there are no adequate preventive or curative treatments for this leading cause of senile dementia, and pharmacological strategies and treatments directed at AD symptoms, and specifically targeted to neurotransmitter deficits, amyloidogenesis, and the progressively inflammatory nature of this brain degeneration, have collectively met with extremely disappointing results (Alzheimer et al., 1995; Hardy, 2006; Lukiw, 2007a, 2008, 2012a; Fisher Center for Alzheimer’s Research Foundation, 2011; Karran et al., 2011; Alzheimer Association, 2012).

## Brain miRNA, Evolutionary Considerations, and Ribonucleotide Sequence Selection for Human Brain miRNA

As is the case for mRNAs, micro RNAs (miRs, miRNAs) are highly developmental stage-, age-, cell-, and tissue-specific. Interestingly, the human brain only uses a highly specific fraction of all known human miRNAs, and of the ∼2000 or so human miRNAs identified to date, the most abundant miRNAs in the human brain probably number less than 40 (Table 1). These 40 prominent species are highly expressed in the human brain and these appear to be highly critical to the regulation of normal, homeostatic brain cell functions. Again, as is the case for mRNAs, astroglial, microglial, neuronal, and other brain cell types each appear to have a different repertoire of miRNA (Sethi and Lukiw, 2009; Li et al., 2012; Table 1).

**Table 1:** **miRNA abundance in the human neocortex – top 80 most abundant miRNAs in control temporal lobe association neocortex (*N* = 18); this group was comprised of a pool of nine males and nine females, mean age 73 ± 7.1 years; post-mortem interval (PMI) of 3 h or less; relative mean abundance as detected by the human MRA-1001 miRNA microfluidic chip analytical platform and giving a raw relative signal yield of ≥2.6 (LC Sciences, Houston, TX, USA)**.

Micro RNA (miRNA; miR)	Relative abundance
hsa-miR-125b-5p	36.6
hsa-miR-26a-5p	30.1
hsa-miR-1273g-3p	28.1
hsa-miR-4324	24.1
hsa-miR-9-3p	24.1
hsa-let-7a-5p	23.5
hsa-miR-9-5p	21.2
hsa-let-7f-5p	20.2
hsa-miR-100-5p	19.6
hsa-miR-26b-5p	19.6
hsa-let-7d-5p	18.4
hsa-let-7c	18.1
hsa-miR-3665	17.8
hsa-miR-29a-3p	17.6
hsa-miR-23b-3p	17.5
hsa-let-7e-5p	15.7
hsa-miR-23a-3p	15.7
hsa-miR-29c-3p	15.2
hsa-miR-128	15.1
hsa-miR-3960	15.0
hsa-miR-125a-5p	14.5
hsa-miR-30b-5p	14.3
hsa-let-7b-5p	14.2
hsa-let-7g-5p	14.2
hsa-let-1185-2-3p	14.1
hsa-miR-4454	13.9
hsa-miR-30c-5p	13.7
hsa-miR-221-3p	12.6
hsa-miR-99a-5p	12.6
hsa-miR-218-5p	12.0
hsa-miR-181a-5p	11.6
hsa-miR-222-3p	11.4
hsa-let-7i-5p	10.8
hsa-miR-23c	10.3
hsa-miR-29b-3p	10.3
hsa-miR-27b-3p	10.2
hsa-miR-27a-3p	9.0
hsa-miR-4787-5p	8.6
hsa-miR-137	8.6
hsa-miR-451a	8.5
hsa-miR-103a-3p	8.4
hsa-miR-107	8.0
hsa-miR-191-5p	7.9
hsa-miR-21-5p	7.9
hsa-miR-151a-5p	7.8
hsa-miR-145-5p	7.7
hsa-miR-151b	7.5
hsa-miR-1915-3p	7.5
hsa-miR-4516	7.5
hsa-miR-124-3p	7.4
hsa-miR-3141	7.3
hsa-miR-99b-5p	7.2
hsa-miR-338-3p	7.1
hsa-miR-195-5p	7.1
hsa-miR-139-5p	6.9
hsa-miR-181b-5p	6.8
hsa-miR-126-3p	6.6
hsa-miR-30a-5p	6.5
hsa-miR-16-5p	6.4
hsa-miR-342-3p	6.3
hsa-miR-5001-5p	6.3
hsa-miR-181d	6.3
hsa-miR-2861	6.1
hsa-miR-30d-5p	5.8
hsa-miR-5100	5.3
hsa-miR-24-3p	5.3
hsa-miR-574-3p	5.2
hsa-miR-638	4.6
hsa-miR-4530	4.3
hsa-miR-4284	4.2
hsa-miR-335-5p	4.1
hsa-miR-92a-3p	4.1
hsa-miR-7-5p	3.6
hsa-miR-361-5p	3.5
hsa-miR-466	3.5
hsa-miR-487b	3.3
hsa-miR-30e-5p	3.1
hsa-miR-101-3p	2.7
hsa-miR-3656	2.6
hsa-miR-3196	2.6
hsa-miR-34a	2.5
hsa-miR-155-5p	1.8*
hsa-miR-146a-5p	0.45*

*hsa-miR-146a and hsa-miR-155 are of relatively low basal abundance in control association neocortex but have been observed to be induced ∼3–25 fold in AD brain (Cui et al., 2010b; Lukiw et al., 2010; Lukiw, 2012a; Lukiw, unpublished observations).

*It should be kept in mind that the evolutionary significance and relevance, if any, of these brain neocortex-abundant miRNAs still needs to be determined, preferably in large, well controlled sample sets. Further, the relative abundance of these miRNAs in other brain anatomical areas, and other factors such as gender effects, individual genotype, and clinical backgrounds, including drug histories, still await to be determined*.

Oligonucleotide and bioinformatics sequence analysis indicates that a 22 nt single stranded small non-coding RNAs (sncRNAs), which is the average size of a typical brain miRNA and composed of four different ribonucleotides, could have over 10^13^ possible sequence combinations, so the fact that there typically much less than about 10^2^ brain-abundant miRNAs suggests a very high developmental and evolutionary selection pressure to utilize only very specific ribonucleotide sequences that will yield biologically useful miRNA-mRNA interactions. As fore-mentioned, miRNAs are highly tissue- and cell-specific, even in adjacent brain cell types (Burmistrova et al., 2007; Pogue et al., 2009; Wang et al., 2010; Arroyo et al., 2011; Madathil et al., 2011). The small size of miRNAs and the identification of miRNA binding proteins that prolong naked miRNA half-life may provide a novel means of regulatory mechanisms “genetic signal storage” or “epigenetic memory” (Pogue et al., 2009; Wang et al., 2010; Arroyo et al., 2011; Madathil et al., 2011). Moreover, the recent discovery of miRNA-containing micro-vesicles further suggests that miRNAs may provide a novel means for paracrine and related forms of inter-cellular, inter-tissue, and perhaps even systemic communication (Wang et al., 2010; Arroyo et al., 2011; Chen et al., 2012; Chen et al., unpublished observations; Vickers and Remaley, 2012). Indeed several extracellular miRNAs have been found to be uniquely stable in plasma, and lipid-based miRNA carriers, in the form of vesicles and lipoprotein particles may be transferred to recipient cells in a paracrine fashion with gene expression changes as a novel form of inter-cellular communication (Chen et al., 2012; Lukiw et al., 2012a,b; Vickers and Remaley, 2012). As for other nuclear transcribed genes, the expression of a cell’s miRNA inventory appears to be regulated by multiple transcription factors, are transcribed as pre-miRNAs, and are not only under the transcriptional control of DNA binding proteins, transcription factors, and RNA polymerase II (RNAPII) and RNAPIII enzymes but further by miRNA-modifying enzymes in the nucleus and cytoplasm that include DGCR8, Exportin-5, Drosha, Dicer, Argonaute, and others (Figure 1; Zhao et al., 2006; Pogue et al., 2009; Wang et al., 2010; Arroyo et al., 2011; Madathil et al., 2011; Chen et al., 2012; Lukiw, 2012b; Vickers and Remaley, 2012).

## Half-Life and Stability of miRNA – Effects of Human Post-Mortem Interval and Total RNA Quality

**Figure 2:**
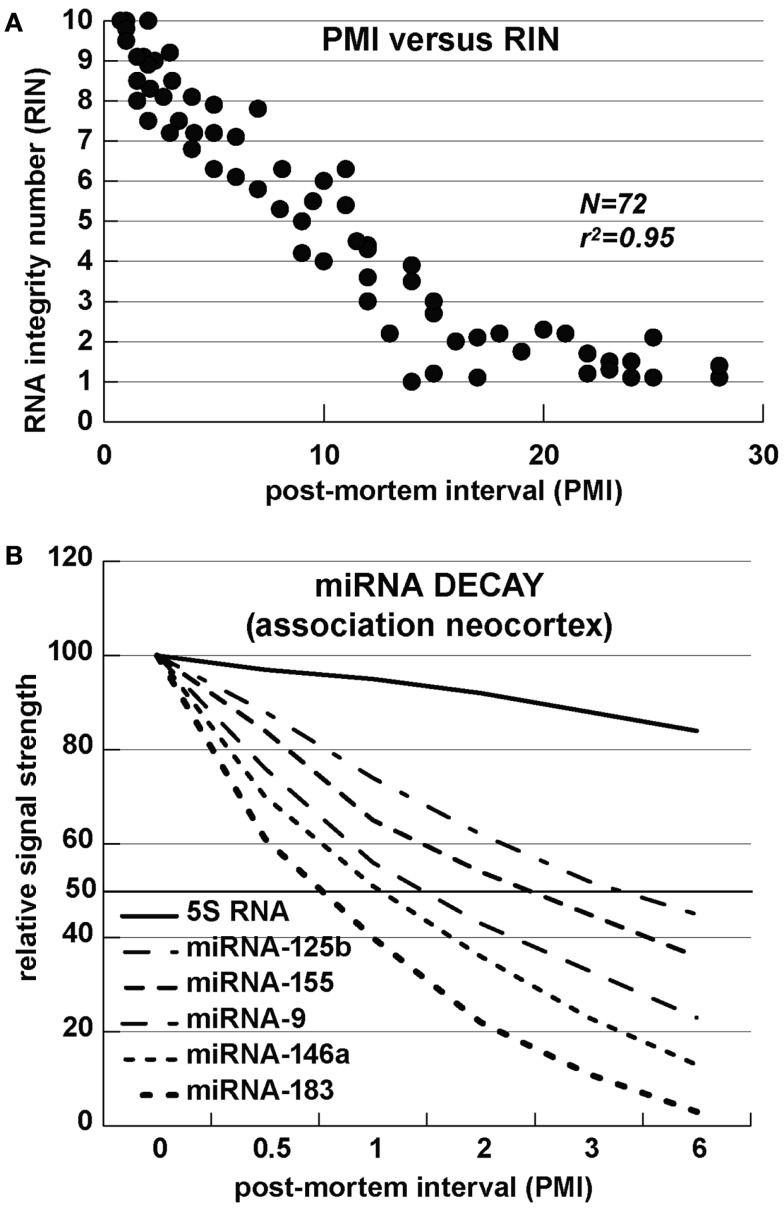
**miRNA stability – (A) RNA integrity number (RIN) versus post-mortem interval (PMI); the correlation of RNA integrity to a positive RIN number is statistically highly significant (*r*^2^ ∼ 0.95; *N* = 72); (B) Half-life of several selected miRNAs; the content, position and 5′–3′orientation of A and U ribonucleotides, and AU-rich elements (AREs) appears to affect miRNA stability, with half-lives generally in the range of 1–7 h (see Sethi and Lukiw, 2009; Rüegger and Großhans, 2012; Zhang et al., 2012)**.

Alzheimer’s disease is uniquely a disease of the human neocortex and occurs in no other mammal or non-human primate. Although amyloid over-expressing transgenic-AD (Tg-AD) murine models are informative, no adequate Tg-AD animal model currently exists that recapitulates all features of AD (Oakley et al., 2006; Ashe and Zahs, 2010; Philipson et al., 2010; Alexandrov et al., 2011; Li et al., 2011a,b). Analysis of miRNA array and Northern-blot-based tracking of specific miRNA abundances and decay kinetics in human neuronal-glial cells in primary co-culture and in short post-mortem interval (PMI, ∼1 h) human brain tissues reveals a limited stability and relatively short half-life for many brain-enriched miRNAs of 3 h or less (Lukiw et al., 1992; Lukiw, 2007b; Sethi and Lukiw, 2009; Cui et al., 2010a; Rüegger and Großhans, 2012; Zhang et al., 2012). The relatively high content of A + U ribonucleotides, AU and UA dinucleotide sequences, and small size in these sncRNAs further support the idea that miRNAs are relatively unstable signaling molecules (Sethi and Lukiw, 2009; Lukiw, 2012b; Rüegger and Großhans, 2012; Zhang et al., 2012). AU-rich elements (AREs) are known predictors of mRNA half-life and, analogous to mRNA stabilities, there appears to be a significant correlation between the rate of miRNA decay and AU + UA dinucleotide content of brain miRNA (*r*^2^ ∼ 0.95, *N* = 72; Chen and Shyu, 1995; Mattick and Makunin, 2005; Mehler and Mattick, 2007; Sethi and Lukiw, 2009). These data therefore suggest that miRNAs may be part of a relatively rapidly deployed signaling system with half-lives (*T_1/2_*) on the order of minutes-to-hours. However these observations should be interpreted with caution as ribonucleotide sequence-specific miRNA binding proteins might be expected to interact with and protect mature miRNAs from impending nucleolytic attack, thus extending their half-lives and adding yet another layer of complexity to miRNA-mediated gene regulation. To date all cumulative data suggests that unless specifically stabilized, certain brain-enriched miRNAs represent a relatively rapidly executed signaling system employing highly transient effectors of central nervous system (CNS) gene expression (Sethi and Lukiw, 2009; Rüegger and Großhans, 2012; Zhang et al., 2012). In past and ongoing studies we have therefore focused only on the analysis of up-regulated miRNAs as down-regulation of miRNA abundance may be, in part, a consequence of their relatively short half-life, and uncontrolled and rapid degradation, especially in human post-mortem tissues with difficult-to-control variability in agonal processes and related effects of dying human brain tissues on biomolecular integrity (Figure 2; Chen and Shyu, 1995; Lukiw et al., 1998, 2012b; Cui et al., 2005; Mattick and Makunin, 2005; Mehler and Mattick, 2007; Sethi and Lukiw, 2009; Lukiw, 2012b; Lukiw and Alexandrov, 2012; Rüegger and Großhans, 2012; Zhang et al., 2012).

## micro RNA – An Overview of a Novel Regulatory Mechanism

As fore-mentioned, robust miRNA-mediated regulation of mRNA complexity in the human CNS is emerging as a critical factor in regulating CNS-specific gene expression during development, plasticity, aging, and age-related neurological disease. As a part of a large and diverse single stranded RNA family in the cell, miRNAs constitute an evolutionarily conserved group sncRNA molecules, and as such represent a recently discovered family of labile, heterogeneous, regulatory sncRNAs (Ambros, 2004; Guo et al., 2010; Taft et al., 2010; Li et al., 2011b). The most prevalent mode of action of miRNAs is the recognition, via base-pair complementarity, with the 3′ untranslated regions (3′-UTRs) of specific mRNA targets (Ambros, 2004; Guo et al., 2010). Depending on 3′-UTR sequence complementarity within the RNA-induced silencing RISC complex, productive miRNA-mRNA interaction results in either reduction or inhibition in the translational efficiency of the target mRNA. Hence a major miRNA-mediated gene control mechanism is that up-regulated miRNAs repress the expression from their mRNA targets (Lukiw et al., 1992; Ambros, 2004; Perron and Provost, 2009; Guo et al., 2010; Taft et al., 2010; Lukiw, 2012b). Indeed ribosome profiling experiments have indicated that mammalian miRNAs predominantly act to decrease the abundance of their target mRNA levels, and in doing so quench specific mRNA transcription to down-regulate expression of that gene (Ambros, 2004; Guo et al., 2010). Although the potential contribution of sncRNA to brain function has been known for at least 20 years (Lukiw et al., 1992), more recently there been a great deal of effort into the molecular and genetic mechanism involving the neurobiological and pathological functions of these miRNAs and sncRNAs in CNS health, development, aging, and disease (Taganov et al., 2006; Burmistrova et al., 2007; Lukiw and Pogue, 2007; Hill et al., 2009; Perron and Provost, 2009; Pogue et al., 2009; Tsitsiou and Lindsay, 2009; Lukiw et al., 2010; Wang et al., 2010; Arroyo et al., 2011; Madathil et al., 2011). *Overall, up-regulated brain miRNAs can therefore be considered a novel neuro-regulatory mechanism that post-transcriptionally down-regulates their target mRNAs, and hence down-regulates selective gene expression in neural cells and tissues*. Multiple different miRNAs can bind to a single mRNA 3′-UTR by a phenomenon known as miRNA convergence; alternately the same single miRNA may recognize multiple mRNA 3′-UTRs by a phenomenon known as miRNA divergence (Lukiw and Alexandrov, 2012). In miRNA divergence, a single miRNA has potential to regulate the expression of a small family of potentially pathogenic genes, as is the case for an up-regulated miRNA-125b targeting the essential synaptic protein synapsin-2 (SYN-2), 15-lipoxygenase (15-LOX), and the cell cycle regulator CDKN2A, or miRNA-146a targeting the innate immune system regulator complement factor H (CFH), interleukin-1 beta associated kinase-1 (IRAK-1), and the βAPP-membrane associated tetraspanin-12 (TSPAN12; Cui et al., 2010a; Li et al., 2011a,b; Lukiw and Alexandrov, 2012; Lukiw et al., 2012a,b). Hence just two up-regulated miRNAs (miRNA-125b) and miRNA-146a can explain many of the pathogenic effects in AD including synaptic and neurotrophic deficits (SYN-2, 15-LOX), astrogliosis (CDKN2A), immune and inflammatory signaling (CFH, IRAK-1), and amyloidogenesis (TSPAN12; Lukiw and Alexandrov, 2012; Lukiw et al., 2012a,b; Figure 2). Overall, significantly up-regulated miRNAs in neurodegenerative disorders such as AD may help explain the large number (roughly 2/3) of all brain gene mRNAs observed to be progressively and significantly down-regulated in anatomical regions sensitive to the AD process (Loring et al., 2001; Colangelo et al., 2002; Lukiw, 2004; Liang et al., 2008; Ginsberg et al., 2012).

**Figure 3:**
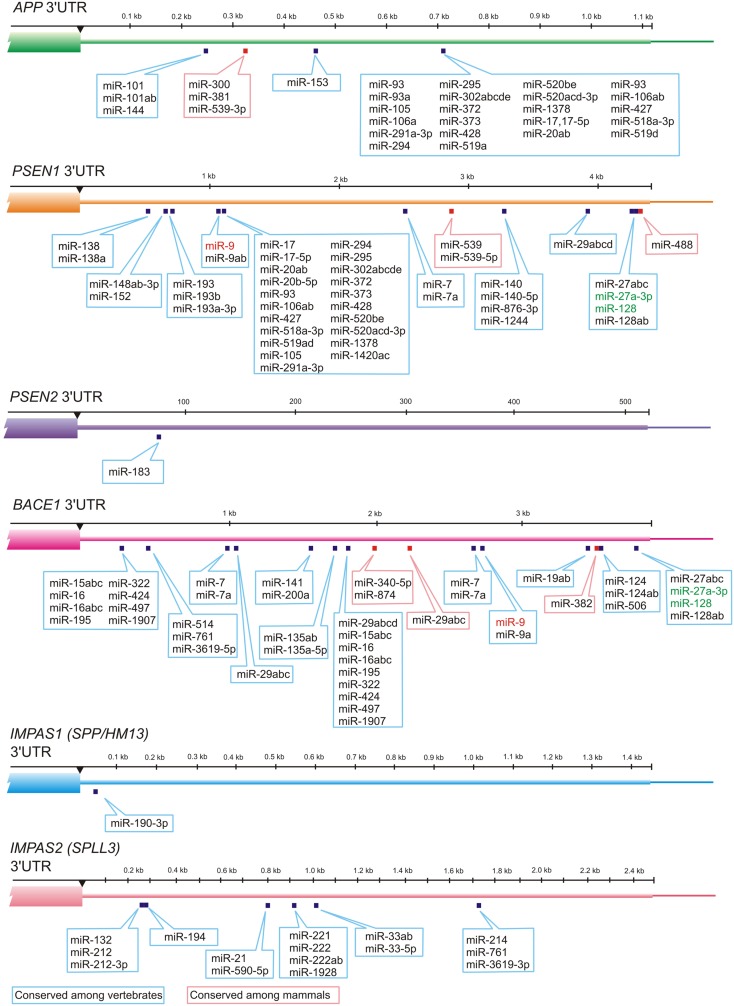
**MiRNAs and their evolutionary-conserved sites in 3′-UTR-gene regions for AD genes (TargetScan prediction, http://www.targetscan.org)**. Presenilin 1 (*PSEN1*) and Presenilin 2 (*PSEN2*) cleave Type I transmembrane proteins (including APP), whereas their distant structural homologs IMPAS1/SPP or other IMPAS/SPPL proteases cleave Type II transmembrane proteins. Interestingly, redundant *PSEN1* and *PSEN2* as well as *IMPAS1* and *IMPAS2* have very different numbers of evolutionary-conserved targets for miRNAs in 3′-UTR. MiRNA-9 that is up-regulated in AD neocortex, hippocampus, and in age-related macular degeneration affected retina is indicated in red. MiRNAs that are most abundant in control temporal lobe association neocortex (see Table 1) are indicated in green.

Numerous miRNAs such as members of miR-15/107 family have ribonucleotide sequence similarity, and therefore have related target mRNAs (Finnerty et al., 2010). Recent studies indicate a possible role for miR-107 in AD pathology. miR-107 is exceptional example of miRNA related to AD, because it has been shown that expression level of the miR-107 can change in brains of AD patients even in the earlier stages of disease (Wang et al., 2008). Moreover miR-107 has a target site in 3′-UTR of β-amyloid cleavage enzyme 1 (*BACE1*) mRNA which is the first enzyme in the amyloidogenic pathway of amyloid precursor protein (APP) processing. miR-107 is enriched in neurons and recent studies indicate that numerous members of miR-15/107 family having the target sites in *BACE1* gene including miR-15a, miR-15b, mR-16, miR-195, miR-103 as well as miR-107; all of these miRNAs are down-regulated in gray matter of AD patients (Wang et al., 2011). Other studies have shown that miR-29a, miR-29b-1, and miR-9 can decrease the expression of *BACE1* (Hebert et al., 2008; Vassar et al., 2009; Shioya et al., 2010). Altogether these data indicate that different miRNAs change the *BACE1* expression level and thus contribute to AD pathology (Figures 2 and 3).

## Human Brain-Abundant miRNAs

Utilization of the most advanced MRA-1001 miRNA microfluidic chip analytical platform currently available (LC Sciences, Houston, TX, USA), about 2000 individual human miRNAs have been identified to date in human cells and tissues. As fore-mentioned, not all of these are present in the brain or in CNS tissues, and the number of most abundant miRNAs in the human brain neocortex number is about 40 (Table 1; Burmistrova et al., 2007; Lukiw, 2012b; Lukiw, unpublished observations). Data in Table 1 were obtained from human brain tissue samples of 3 h or less and were compared to age-matched controls in the same brain anatomical region that is targeted by AD. Although we cannot exclude the contribution to AD pathology of other human brain miRNAs, the inducible, NF-kB-regulated miRNAs miRNA-9, miRNA-125b, miRNA-146a, and miRNA-155 show some of the most significant up-regulated changes in all AD brains so far examined (*N* = 72; Lukiw et al., 2008; Cui et al., 2010a; Lukiw, 2012b). The following section gives further structural and functional details on these inducible miRNAs – including miRNA-9, miRNA-125b, miRNA-146a, and miRNA-155 – that have been shown to be up-regulated in short PMI Alzheimer hippocampus and/or neocortex.

## Up-Regulated NF-κB-Sensitive miRNAs in AD Neocortex, Hippocampus, and in Age-Related Macular Degeneration Affected Retina

### miRNA-9

The 23 oligonucleotide human miRNA-9 (GenBank NR_029691.1; chr 1q22) is an NF-kB-regulated, retinoic acid-inducible sncRNA enriched in primary human neural cells (a neuronal-glial cell primary co-culture), in cultured human NTera-2 clone D1 cells and in human ARPE-19 cells (Sempere et al., 2004; Du and Zamore, 2005; Singh, 2007; Singh, unpublished observations; Williams et al., 2008; Sethi and Lukiw, 2009). In multiple independent studies miRNA-9 has been associated with neurogenesis, morphogenesis, developmental patterning, brain cell proliferation, and glioblastoma (Sempere et al., 2004; Singh, 2007; Bazzoni et al., 2009; Hébert and De Strooper, 2009; Tsitsiou and Lindsay, 2009; Arora et al., 2010; Kutty et al., 2010a,b; Zhang et al., 2010; Bonev et al., 2011; Shibata et al., 2011; Yuva-Aydemir et al., 2011). miRNA-9 is easily detectable in both the neocortex and retina it was found to be one of the most variably abundant miRNA in these tissues, perhaps indicative of its highly inducible nature (Singh, 2007; Bazzoni et al., 2009; Arora et al., 2010; Arora et al., unpublished observations; Kutty et al., 2010a,b; Shibata et al., 2011; Yuva-Aydemir et al., 2011). Enriched in AU or UA dinucleotide content, with a half-life of only 30–60 min in both cultured human primary brain cells and human brain tissues, miRNA-9 is a brain and retinal abundant miRNA possessing the shortest half-life of any miRNA studied to date (Williams et al., 2008; Pogue et al., 2009; Sethi and Lukiw, 2009). Interestingly, miRNA-9 exerts significant control of neural progenitor cell proliferation and differentiation in the developing telencephalon by regulating the expression of multiple transcription factors including Foxg1, Nr2e1, and Pax6 (Shibata et al., 2011). We further note that miRNA-9 is developmentally regulated and abundant in ARPE-19 retinal epithelial cells, and decreases in expression as human brain and retinal cells age in primary culture, in accordance with its established role as a developmentally regulated miRNA (Arora et al., 2010; Kutty et al., 2010a,b; Yuva-Aydemir et al., 2011). Knockdown of miRNA-9 in neural progenitor cells, results in an inhibition of neurogenesis along the anterior-posterior axis of the CNS, and miRNA-9 is also significantly down-regulated in the tissues of fetuses with severe congenital abnormalities, including anencephaly (Zhang et al., 2010; Bonev et al., 2011). In a recent miRNA analysis of whole murine globes using both microarray and RNA *in situ* hybridization procedures, miRNA-9 expression was restricted to the retina and not seen in the cornea, lens, iris, or ciliary body (Hébert and De Strooper, 2009; Tsitsiou and Lindsay, 2009).

### miRNA-125b

One of the most intensively studied, and human brain and retinal abundant miRNA is the inducible miRNA-125b (GenBank NR_029671.1; chr 11q24.1; Table 1). miRNA-125 was first shown to be up-regulated in differentiating mouse and human neurons, and since implicated in mammalian neuronal development and function in the brain (Madathil et al., 2011). Both miRNA-125b and miR-9 play central roles in neuronal differentiation during retinal development (Arora et al., 2010; Kutty et al., 2010a,b; Maiorano and Hindges, 2012). The abundance of miRNA-125b has been shown to be significantly induced by neurotoxic metal sulfates that generate robust levels of oxidative stress, and miRNA-125b is also up-regulated in brain cancers where it apparently targets and down-regulates CDKN2A, a negative regulator of cell growth (Sempere et al., 2004; Lukiw, 2007a,b; Sethi and Lukiw, 2009; Pogue et al., 2010; Feng et al., 2011). Up-regulated miRNA-125b further associates with glial cell proliferation and astrogliosis in inflammatory neurodegenerative conditions such as AD and Down’s syndrome (DS), as well as in glioma and glioblastoma multiforme (Pogue et al., 2010; Feng et al., 2011; Lukiw et al., 2011). Up-regulation of miRNA-125b is further associated with down-regulation of both the 15-lipoxygenase (15-LOX) and the synaptic vesicle-associated phosphoprotein synapsin-2 (SYN-2; Lukiw et al., 2008, 2011; Feng et al., 2011; Pogue et al., 2011; Li et al., 2012; Lukiw and Alexandrov, 2012). The 15-LOX enzyme is essential in the conversion of the essential omega-3 fatty acid docosahexaenoic acid (DHA) into the potent neuroprotectin D1 (NPD1), and deficits in 15-LOX correlate with NPD1 deficits in AD brain and other human tissues (Lukiw et al., 2005; Hennig et al., 2007; Zhao et al., 2011; Chen et al., 2012). The neuronal-enriched phosphoprotein SYN-2 that associates with the cytoplasmic surface of synaptic vesicles is also a miRNA-125b target (Yao et al., 2003). MiRNA-125b up-regulation is further associated with SYN-2 down-regulation in inflammatory neuro-degeneration and CFH down-regulation in human primary astroglial cells (Yao et al., 2003; Pogue et al., 2010, 2011). Interestingly miRNA-125b and miRNA-146a have tandem binding sites in the mRNA 3′-UTR of human CFH, a key repressor of the innate and immune response in the brain; indeed miRNA-125b and miRNA-146a appear to be involved in immune system activation, at least in part by reducing interferon regulatory factor 4 (IRF4) levels thus potentiates induction of the immune response (Cui et al., 2010a; Chaudhuri et al., 2011).

### miRNA-146a

miRNA-146a (GenBank NR_029701; chr 5q34) was first described as an inducible, NF-κB-regulated pro-inflammatory miRNA that was found to target signaling proteins of innate immune responses, and more specifically the 232 nt 3′-UTR of CFH mRNA in human monocytes (Taganov et al., 2006, 2007; Baltimore et al., 2008; Lu et al., 2010). More recently nt analysis of the CFH mRNA 3′-UTR indicates that the CFH mRNA 3′-UTR contains multiple binding sites for miRNA-9, miRNA-125b, miRNA-146a, and miRNA-155 including an overlapping miRNA regulatory control (MiRC) region that contains overlapping binding sites for miRNA-146a and miRNA-155 (Lukiw et al., 2012a,b). Interestingly the brain and retina may use alternate miRNA binding sites in the CFH mRNA 3′-UTR to regulate CFH expression, and this may have a bearing on innate immune and inflammatory responses to stress in AD or age-related macular degeneration (AMD; Lukiw et al., 2012a,b). Elevated miRNA-146a in AD brain has been shown to also specifically target CFH and the interleukin-1 associated kinase-1 (IRAK-1) mRNAs, and is believed to contribute to altered innate immune responses and neuroinflammation in degenerating human brain cells and tissues in inflammatory neurodegenerative diseases including AD, AMD, prion disease, in experimental and human temporal lobe epilepsy, in experimental diabetes in retinal microvessel endothelial cells, and in primary human brain cells stressed with ROS-generating metal sulfates (Lukiw and Pogue, 2007; Lukiw et al., 2008, 2011; Pogue et al., 2009, 2011; Aronica et al., 2010; Cui et al., 2010a; Li et al., 2011a,b, 2012; Madathil et al., 2011; Lukiw and Alexandrov, 2012; Saba et al., 2012). Although CFH was classically regarded as highly abundant human serum protein of hepatic origin, abundant CFH presence in brain and retinal tissues suggests CFH involvement in the innate immune response and inflammatory regulation within the “privileged immunology” of these tissues (Lukiw, 2007a; Lukiw et al., 2008; Ashe and Zahs, 2010; Donoso et al., 2010; Gehrs et al., 2010; Taft et al., 2010; Zipfel et al., 2010; Deangelis et al., 2011; Fisher Center for Alzheimer’s Research Foundation, 2011; Kondo et al., 2011; Alzheimer Association, 2012). While miRNA-146a is the least basally abundant miRNA when compared to miRNA-9, miRNA-125b, and miRNA-155, it is the most inducible and up-regulated miRNA in human neuronal and astroglial cells compared to all other NF-κB-regulated miRNA species so far indentified (Lukiw et al., 2008, 2011; Cui et al., 2010a; Pogue et al., 2011; Lukiw, 2012a,b). Interestingly, miRNA-146a may be the most induced of all of the up-regulated miRNAs in AD and prion brain due to the presence of three tandem, canonical NF-κB binding sites within the human pre-miRNA-146a promoter (Lukiw et al., 2008, 2012b).

### miRNA-155

A cytokine, NF-κB, and cell cycle-regulated miRNA, miRNA-155 (GenBank NR_030784; chr 21q21.3) is also abundant in the human neocortex and retina, and has approximately 45% sequence homology to miRNA-146a (O’Connell et al., 2007; Lukiw and Alexandrov, 2012; Lukiw et al., 2012a,b). Interestingly, miRNA-146a and miRNA-155 have partially overlapping binding (recognition) sites in the CFH mRNA 3′-UTR, and miRNA-146a and miRNA-155 together define an overlapping MiRC region in the CFH 3′-untranslated region (3′-UTR; 5′-TTTAGTATTAA-3′) herein either of these miRNAs may interact (Lukiw and Alexandrov, 2012). As is true for miRNA-146a, inflammatory cytokines increase miRNA-155 expression in human retinal pigment epithelial cells by activation of the JAK/STAT signaling pathway (O’Connell et al., 2007). Several additional studies indicate that miRNA-146a and miR-155 together play a key role in regulating several critical pathways that orchestrate innate immune responses and chronic inflammatory processes that are conserved across many different human tissue systems; this may be a consequence of their related ribonucleotide sequence (O’Connell et al., 2007; Tsitsiou and Lindsay, 2009; Kutty et al., 2010a,b; Roy and Sen, 2011).

### miRNA-9, miRNA-125b, miRNA-146a, and miRNA-155 mRNA targets in the brain

**Figure 4:**
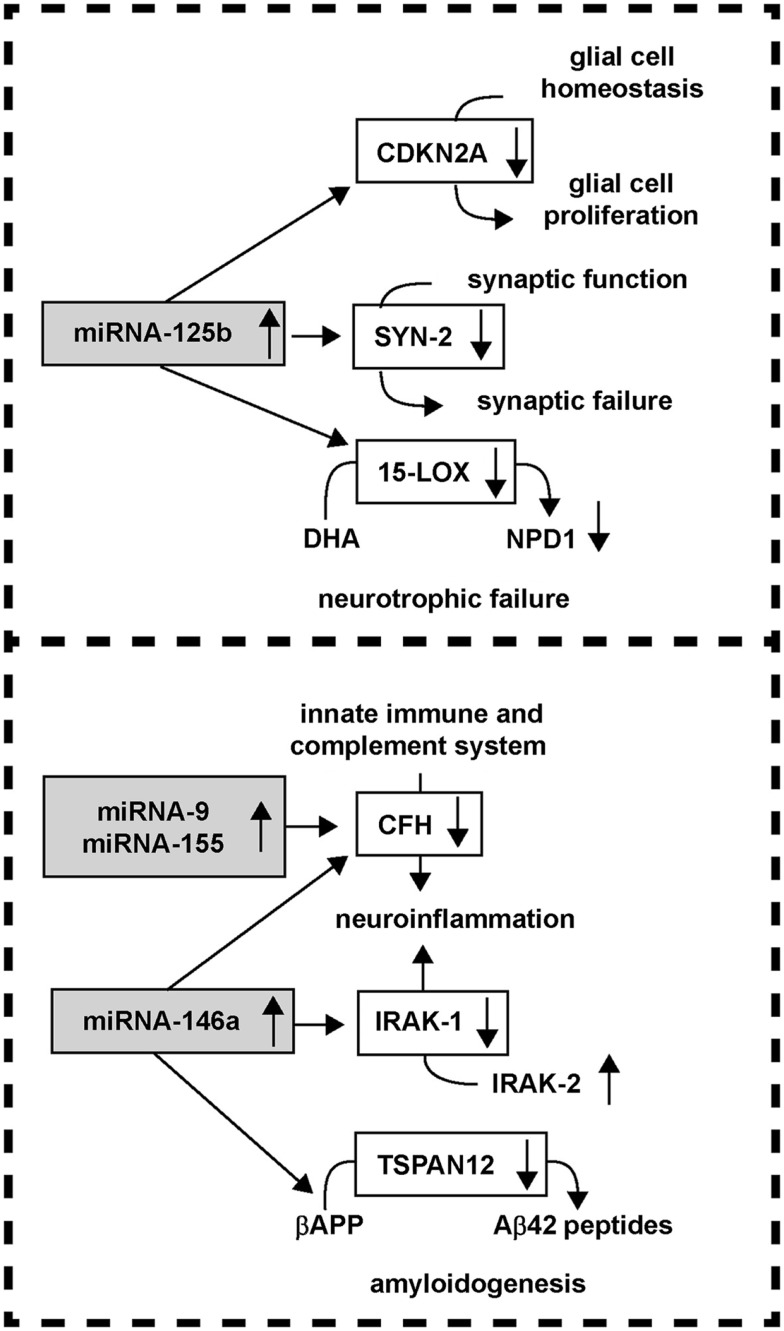
**miRNA up-regulation and down-regulation of AD-relevant gene expression; a relatively small number of up-regulated miRNAs may impact several key pathological features of AD, including the modulation of glial cell proliferation, synaptic and neurotrophic failure, neuroinflammation, and amyloidogenesis**.

The actions of up-regulated miRNAs on down-regulating their target mRNAs in AD is attractive in that many interwoven aspects of AD neuropathology and progression can be explained by the pathogenetic activities of a relatively small number of these sncRNAs. For example, up-regulation of the human brain-abundant miRNA-125b is associated with down-regulation in the expression of the cyclin-dependent kinase inhibitor 2A (CDKN2A), a miRNA-125b target, and negative regulator of astroglial cell growth (Pogue et al., 2010). A strong positive correlation between miRNA-125b abundance and the glial cell markers glial fibrillary acidic protein (GFAP) and vimentin, and CDKN2A down-regulation is apparent in advanced AD and in DS brain; both of these chronic neurological disorders are typified by a progressive astrogliosis (Pogue et al., 2010; Lukiw and Alexandrov, 2012). The neuronal-enriched phosphoprotein SYN-2 that associates with the cytoplasmic surface of synaptic vesicles is also a miRNA-125b target, and miRNA-125b up-regulation also correlates with SYN-2 down-regulation in AD brain (Yao et al., 2003; Lukiw and Alexandrov, 2012). Another miRNA-125b target is 15-lipoxygenase (15-LOX; Lukiw et al., 2005; Hennig et al., 2007; Zhao et al., 2011; Lukiw and Alexandrov, 2012). The 15-LOX enzyme is essential in the conversion of the essential omega-3 fatty acid DHA into the potent docosanoid neuroprotectin D1 (NPD1), and deficits in 15-LOX significantly correlate with NPD1 deficits in AD brain (Lukiw et al., 2005; Hennig et al., 2007; Zhao et al., 2011). Hence, the actions of miRNA-125b divergence can explain three characteristic deficits in AD brain including glial cell proliferation, synaptic, and neurotrophic failure and other key pathophysiological aspects of the AD process (Figure 4; Yao et al., 2003; Lukiw et al., 2005; Hennig et al., 2007; Taganov et al., 2007; Baltimore et al., 2008; Lu et al., 2010; Pogue et al., 2010; Chaudhuri et al., 2011; Zhao et al., 2011; Lukiw and Alexandrov, 2012).

In line with these ideas, CFH is a key negative regulator of the innate immune and complement system, and miRNA-9, miRNA-146a, and miRNA-155 up-regulation is associated with decreased CFH in the neuro-degeneration associated with AD, prion disease, and temporal lobe epilepsy (Lukiw et al., 2008; Hébert and De Strooper, 2009; Perron and Provost, 2009; Aronica et al., 2010; Li et al., 2010; Saba et al., 2012). An up-regulated miRNA-146a is further associated with down-regulation of the interleukin-1β-associated kinase-1 (IRAK-1), with a compensatory surge in IRAK-2 that is involved in a more chronic and sustained form of NF-kB activation that supports pro-inflammatory signaling in human brain cells (Granic et al., 2009; Kaltschmidt and Kaltschmidt, 2009; Cui et al., 2010a). Additionally, the mRNA encoding a four-time membrane spanning integral membrane protein tetraspanin-12 (TSPAN12) is also a target for miRNA-146a, and up-regulated miRNA-146a further contributes to the down-regulation of TSPAN12 as is observed in AD brain and in cytokine and Aβ-peptide-stressed human brain cells (Junge et al., 2009; Xu et al., 2009; Li et al., 2011a,b). Interestingly, sufficient TSPAN12 appears to be required for the neurotrophic processing of the beta-APP into a neurogenic soluble form of beta-APP known as sAPPα; insufficient TSPAN12 is associated with the induction of amyloidogenesis, vascular aberrations, and Aβ42 peptide evolution (Junge et al., 2009; Xu et al., 2009). Therefore, the integrated miRNA-mRNA interactions of as few as two induced human brain miRNAs – miRNA-125b and miRNA-146a – may in part explain not only the observed down-regulation of CDKN2A, SYN-2, 15-LOX, CFH, IRAK-1, and TSPAN12 expression but may also contribute to pathogenic deficiencies linked with astrogliosis, synaptogenesis, neurotrophic support, innate immune and inflammatory signaling, and amyloidogenesis in the core mechanism of the AD process.

## Potential Reversal of Pathogenic miRNA Actions Using Anti-miRNA Strategies

A growing body of evidence suggests that antagonizing miRNA activity through the use of anti-miRNAs (AMs, antagomirs) may be a useful approach to quenching the pathogenic effects of up-regulated miRNAs in the brain (Lukiw et al., 2008; Cui et al., 2010a; Lukiw, 2012b). Specifically designed synthetic AMs, perfectly antisense to naturally occurring miRNAs, may have their half-life extended through the use of various strategies including locked nucleic acid (LNA) protection. LNA-modified AMs have been shown to be highly efficacious in human brain cells in primary culture but the implementation of these technologies in human clinical trials awaits further development (Lukiw, 2012a).

## Conclusion

The conservation and extreme selection of specific miRNAs among diverse species suggests that they bear important and conserved biological functions throughout evolution (Rogaev, 2005; Weber, 2005; Wang et al., 2010). Indeed, accumulating data indicate that miRNAs are playing an expanding role from neuro-regeneration to neuro-degeneration in human brain development and neurological disorders (Rogaev, 2005; Lukiw and Pogue, 2007; Lukiw et al., 2008, 2011; Pogue et al., 2011; Li et al., 2012; Lukiw and Alexandrov, 2012). The characterization of RNA complexity in short PMI human brain tissues is highly informative, even though miRNA stability may be a real concern in data and bioinformatics analysis (Lukiw et al., 1992, 1998; Cui et al., 2005, 2010a; Lukiw, 2007b; Lukiw and Pogue, 2007; Sethi and Lukiw, 2009; Rüegger and Großhans, 2012; Zhang et al., 2012). Interestingly, whole nuclei isolated from human brain tissues with PMI’s of up to 3–4 h efficiently support run-on gene transcription, an effect that is inhibited using the octapeptide RNAPII nt incorporation and translocation inhibitor α-amanitin (Lukiw et al., 1998; Cui et al., 2005; Lukiw and Pogue, 2007). The contribution of several brain cell types in CNS tissues to miRNA speciation, abundance, and complexity needs to be further addressed by analyzing individual human brain cell types in primary cell culture, in Tg-AD models and perhaps by using single cell amplification techniques (Pogue et al., 2010; Li et al., 2011a,b; Ginsberg et al., 2012; Saba et al., 2012). Analysis of miRNA in autopsied brain tissues with PMIs many-fold greater than the half-lives of individual miRNAs may lead to inaccurate conclusions concerning their absolute abundance, and hence the contribution of specific miRNAs to gene regulation in the brain during development, aging, and in disease processes (Table 1, Figure 1). To add another layer of complexity, it should also be noted that different brain cells from different brain regions may have different stabilities for the same sncRNAs and miRNAs due to initial abundance, the stabilizing effects of miRNA binding proteins, compartmentalization, relative mean abundances, and related intrinsic factors such as miRNA secondary and tertiary structure and miRNA binding proteins (Lukiw et al., 1998, 2008; Cui et al., 2005; Sethi and Lukiw, 2009; Feng and Feng, 2011; Im and Kenny, 2012; Rüegger and Großhans, 2012; Zhang et al., 2012).

## Summary

Human brain miRNAs are currently acquiring essential and determinant roles in advancing our understanding of the regulation of CNS-relevant gene expression and the epigenetic regulatory mechanisms of aging in both health and disease (Lukiw et al., 1992, 2010, 2012b; Ambros, 2004; Oakley et al., 2006; Lukiw, 2007b; Perron and Provost, 2009; Pogue et al., 2009; Sethi and Lukiw, 2009; Tsitsiou and Lindsay, 2009; Ashe and Zahs, 2010; Cui et al., 2010a; Guo et al., 2010; Philipson et al., 2010; Alexandrov et al., 2011; Li et al., 2011b; Madathil et al., 2011). That approximately 85% of eukaryotic genomes are transcribed into various kinds of RNA, and that only about 2% of the genome is transcribed into protein, underscores the potential of various complex layers of RNA signaling and RNA-based regulatory mechanisms that are known to contribute to homeostatic gene control (Oakley et al., 2006; Taganov et al., 2006; Lukiw, 2007a,b). It will be interesting to further research the complimentary roles of specific transcription factors and miRNAs, and the interactive roles of these sncRNAs in the phenomenon of miRNA divergence and convergence, as they appear to play highly interrelated and cooperative roles in the regulation of CNS gene expression (Burmistrova et al., 2007; Lukiw, 2007b; Ashe and Zahs, 2010; Cui et al., 2010a; Philipson et al., 2010; Wang et al., 2010; Arroyo et al., 2011). To cite just one example, pre-miRNA-146a, an inducible, NF-κB-regulated gene, has been associated with the development and propagation of prion disease, epileptogenesis, viral infection, neuro-degeneration, and the induction of neuro-inflammatory signaling and altered innate immune responses in primary human neural cell culture models of AD (Lukiw, 2007b, 2008; Perron and Provost, 2009; Sethi and Lukiw, 2009; Ashe and Zahs, 2010; Lukiw et al., 2010). It may be possible to target these pathogenic miRNA effects in AD and related neurological disorders using AM (antagomir) strategies, either alone or in combinatorial approaches (Lukiw et al., 2008, 2010; Pogue et al., 2009; Tsitsiou and Lindsay, 2009; Cui et al., 2010a; Madathil et al., 2011). Overall, selectively up-regulated miRNA abundances in regions of brain targeted by AD neuropathology, that are not increased in the same brain anatomical regions in normal aging or in other neurological disorders, further implicates their selective involvement in AD pathology and the pathogenetic and regulatory signaling processes associated with multiple interwoven aspects of the neurodegenerative disease process (Oakley et al., 2006; Taganov et al., 2006; Lukiw, 2007b; Guo et al., 2010). A clearer understanding of how miRNAs influence the initiation and progression of AD and related neuropsychiatric disorders may not only reveal fundamental insights into the causes of these devastating human neurological disorders, but should also provide novel pharmacological strategies for advanced and efficacious intervention in the future clinical management of AD (Feng and Feng, 2011; Im and Kenny, 2012; Lukiw, 2012a; Percy et al., 2012).

## Conflict of Interest Statement

The authors declare that the research was conducted in the absence of any commercial or financial relationships that could be construed as a potential conflict of interest.
